# Ketamine normalizes high-gamma power in the anterior cingulate cortex in a rat chronic pain model

**DOI:** 10.1186/s13041-020-00670-w

**Published:** 2020-09-23

**Authors:** Isabel D. Friesner, Erik Martinez, Haocheng Zhou, Jonathan Douglas Gould, Anna Li, Zhe Sage Chen, Qiaosheng Zhang, Jing Wang

**Affiliations:** 1 Department of Anesthesiology, Perioperative Care and Pain, New York University School of Medicine, New York, NY 10016 USA; 2 Department of Psychiatry, New York University School of Medicine, New York, NY 10016 USA; 3 College of Arts and Sciences, New York University, New York, NY 10003 USA; 4 Department of Neuroscience & Physiology, New York University School of Medicine, New York, NY 10016 USA; 5 Neuroscience Institute, New York University School of Medicine, New York, NY 10016 USA

**Keywords:** Chronic pain, Anterior cingulate cortex, Ketamine, Gamma band power, Local field potential

## Abstract

Chronic pain alters cortical and subcortical plasticity, causing enhanced sensory and affective responses to peripheral nociceptive inputs. Previous studies have shown that ketamine had the potential to inhibit abnormally amplified affective responses of single neurons by suppressing hyperactivity in the anterior cingulate cortex (ACC). However, the mechanism of this enduring effect has yet to be understood at the network level. In this study, we recorded local field potentials from the ACC of freely moving rats. Animals were injected with complete Freund’s adjuvant (CFA) to induce persistent inflammatory pain. Mechanical stimulations were administered to the hind paw before and after CFA administration. We found a significant increase in the high-gamma band (60–100 Hz) power in response to evoked pain after CFA treatment. Ketamine, however, reduced the high-gamma band power in response to evoked pain in CFA-treated rats. In addition, ketamine had a sustained effect on the high-gamma band power lasting up to five days after a single dose administration. These results demonstrate that ketamine has the potential to alter maladaptive neural responses in the ACC induced by chronic pain.

## Introduction

Chronic pain impacts around 20% of people globally [1]. Current treatments cause many side effects due to the incomplete understanding of the mechanisms of chronic pain [2]. For example, there has been a dramatic rise in prescription of opioid drugs to treat chronic postoperative pain [3–5], which has in turn lead to sedation, respiratory depression, dependence and even addiction [5, 6]. Therefore, there is an urgent need not only to propel our understanding of the mechanism of chronic pain, but also to find alternative effective analgesics with minimal side effects [7].

Recently, administrations of low, sub-anesthetic doses of ketamine either alone or as an adjunct to opioid therapies have been shown to be an effective treatment in postoperative and acute pain settings [8–12]. In addition, ketamine has long been safely used as an general anesthetic and analgesic agent [13]. Recently, the FDA approved a new intranasal preparation of ketamine for the treatment of acute depressive symptoms [14]. Specifically regarding chronic pain states, human and animal studies have shown the effectiveness of ketamine injections in eliciting a rapid response to peripheral nociceptive inputs, and in some cases, showing sustained analgesic efficacy lasting up to a week [15–18]. These fact-acting and persistent effects of ketamine prompt further investigation into its analgesic mechanisms.

Recent studies have shown chronic pain could impair the balance between sensory and affective components of pain, leading to a generalized, anatomically nonspecific enhancement in the aversive response to acute noxious stimuli [19–21]. In addition, chronic pain alters synapses and circuits in the cerebral cortex and contributes to abnormalities in pain affect [22–24]. The anterior cingulate cortex (ACC) has been known to modulate the affective-motivational component of pain and lead to hyperactivity of neuronal components in response to pain [3, 7, 24–26]. The ACC mediates the aversive response to pain evoked by noxious stimulations.[4, 26–28] These findings have been replicated in other model systems, including rabbits and monkeys, where increases in extracellular activity in the ACC have been observed to occur in response to stimulations in freely moving animals and humans [29–32]. Furthermore, studies have shown that ACC neurons undergo synaptic plasticity in chronic pain conditions [7, 25, 33]. Thus, there is a possibility that neuronal responses to noxious stimuli in the ACC may show distinct patterns in the chronic pain state.

Ketamine has been shown to affect multiple areas of the brain, including the ACC [34, 35]. Our previous animal study showed that a single dose of ketamine was able to inhibit this abnormal enhancement in pain aversion for 5 days in the ACC [36]. *N*-Methyl-D-aspartate receptors (NMDARs) are glutamate receptors that play a role in the excitatory response of the central nervous system.[37, 38] In a chronic pain state, changes in neuroplasticity lead to excessive activation of NMDARs [39, 40]. As a NMDARs antagonist, ketamine likely influences the affective-motivational component through regulating neuroplasticity [41, 42]. However, the mechanism of this enduring effect has yet to be widely understood since glutamate is a broadly distributed neurotransmitter in the brain. Recently, we observed a reduction in the hyperactivity of ACC neurons in chronic pain states after ketamine administration, allowing individual neuron to return their spiking rate in response to acute noxious stimuli back to baseline levels [36].

In contrast to action potentials (or spikes), local field potentials (LFPs) measure collective behavior of ensemble neurons, and frequency-specific LFPs are thought to process distinct network information. For instance, the high-gamma (60–100 Hz) oscillatory activity is often seen to correlate with spike synchrony and could be utilized as a substitute for assessing output of neuronal activity. Moreover, neural oscillations in broadband power spectrum have been shown to be involved in neuropathic pain, characteristics of which can be utilized to analyze aspects of pain and pain relief [43]. Thus, we hypothesize that ketamine can rescue or reverse enhanced high-gamma band power in the ACC in response to noxious stimuli in the chronic pain condition.

In this study, we recorded LFPs in the ACC in freely moving rats from a classic chronic pain model—complete Freund’s adjuvant (CFA) model of inflammatory pain. Mechanical pinprick was used to generate nociceptive inputs, and LFP spectral powers were evaluated before and after ketamine treatment. Interestingly, we found chronic pain increased the high-gamma band power in ACC neurons. A single sub-threshold anesthetic dose of ketamine, however, reversed high-gamma enhancement in response to evoked pain in the chronic pain condition. Therefore, our results support that ketamine could rescue chronic pain induced neuroplasticity at a network level.

## Results

### Elevation in the high-gamma band power in the ACC in response to chronic pain

**Fig. 1:**
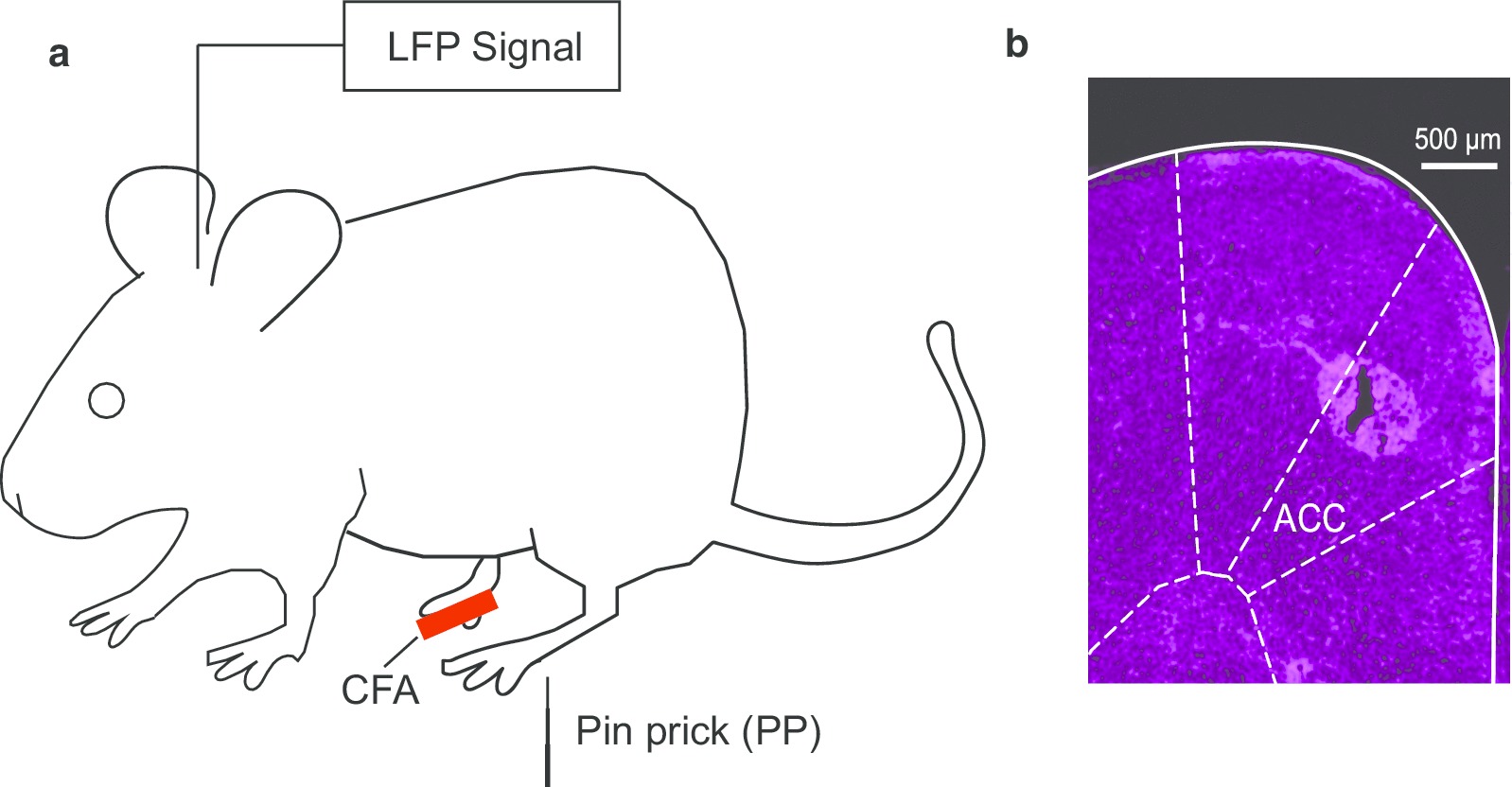
Experimental Design. **a** Pinprick stimulations were conducted in the uninjured paw contralateral to the CFA injection. Local field potentials (LFPs) were recorded from the anterior cingulate cortex (ACC) contralateral to peripheral stimulations. **b** Location of recording electrodes in the ACC

**Fig. 2:**
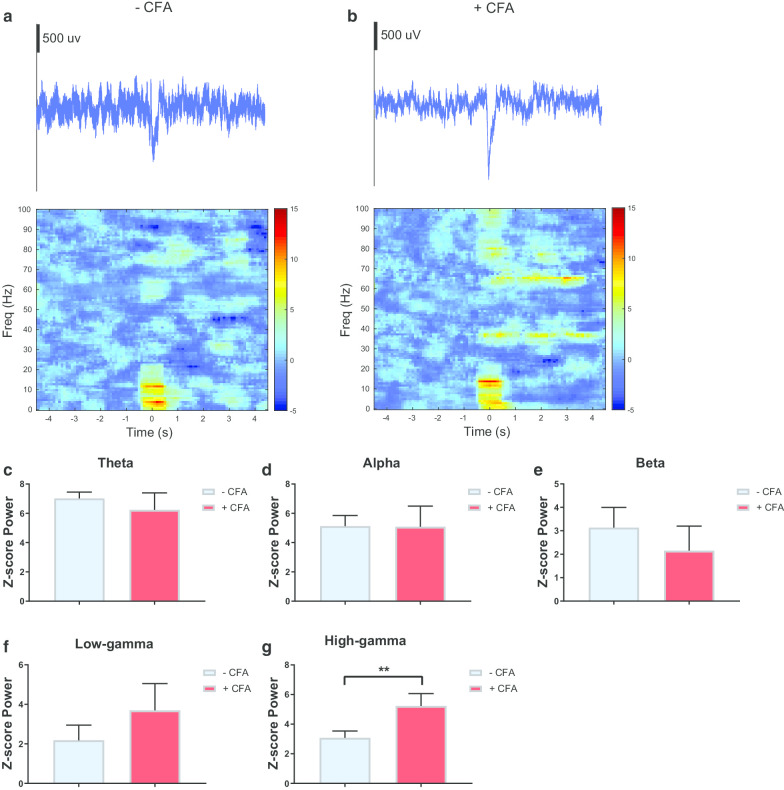
Chronic pain increased high-gamma band power in the ACC. **a** Example of trial-averaged LFP raw trace, in the naïve condition. The panel directly below the raw trace shows trial-averaged time–frequency spectrum, with time 0 denoting the time of pinprick stimulation. **b** Example of trial-averaged LFP raw trace, post CFA injection. The panel directly below the raw trace shows trial-averaged time–frequency spectrum, with time 0 denoting the time of pinprick stimulation. **c** Z-score power in the theta frequency band (4–8 Hz) before and after CFA injection. **d** Z-score power in the alpha frequency band (8–15 Hz) before and after CFA injection. **e** Z-score power in the beta frequency band (15–30 Hz) before and after CFA injection. **f** Z-score power in the low-gamma frequency band (30–60 Hz) before and after CFA injection. **g** Z-score power in the high-gamma frequency band (60–100 Hz) before and after CFA injection (p = 0.0084, Paired t-test). Error bars represent SEM. *p < 0.05, **p < 0.01

Extracellular activities (spikes and LFPs) were recorded from chronically implanted tetrodes in the ACC region of awake, freely moving rats (Fig. 1a, b). Multi-channel LFP data were collected over multiple sessions, each consisting of continuous recordings over at least 30 noxious pinprick stimulations to the rat’s hind paw, contralateral to the ACC tetrode implants, though a mesh table (Fig. 1a). Single-trial LFP raw data were collected and averaged to produce trial-averaged LFP raw traces, as single-trial neural signals can be very noisy. The trial-averaged LFP raw data were then used to generate spectrograms, and we analyzed the broadband power spectra (4–100 Hz, “all frequency” with the exception of low-frequency (1–3 Hz) bands, which often contain artifacts). In the pre-CFA state, there was minimal activity in the gamma band as seen in the trial-averaged LFP spectrogram (Fig. 2a).

Next, we induced chronic pain by injecting complete Freund’s adjuvant (CFA) to the rat’s hind paw, ipsilateral to the ACC recording sites [44]. In contrast to rats in the naïve state, CFA-injected rats demonstrated a noticeable increase in the activity in the high-gamma region as shown in the trial-averaged LFP spectrogram (Fig. 2b). Furthermore, as demonstrated by the trial-averaged LFP raw traces, the peak in the chronic pain state is greater than the peak in the naïve state (Fig. 2a, b). We quantitated the LFP power in the naïve and chronic pain conditions, and compared power in the theta (4–8 Hz), beta (8–15 Hz), alpha (15–30 Hz), low-gamma (30–60 Hz), and high-gamma (60–100 Hz) bands. There was no statistical significance between the naïve and chronic pain conditions in the theta, alpha, beta, and low-gamma bands (Fig. 2c–g). However, there was a statistically significant difference in the high-gamma band, demonstrating an increase in power in the high-gamma band in CFA-treated rats (p = 0.0084, Paired t-test, Fig. 2h). The data indicates that chronic pain induces increased LFP activity and power in the high-gamma band.

### A single low dose of ketamine reduces the LFP power in the ACC

**Fig. 3:**
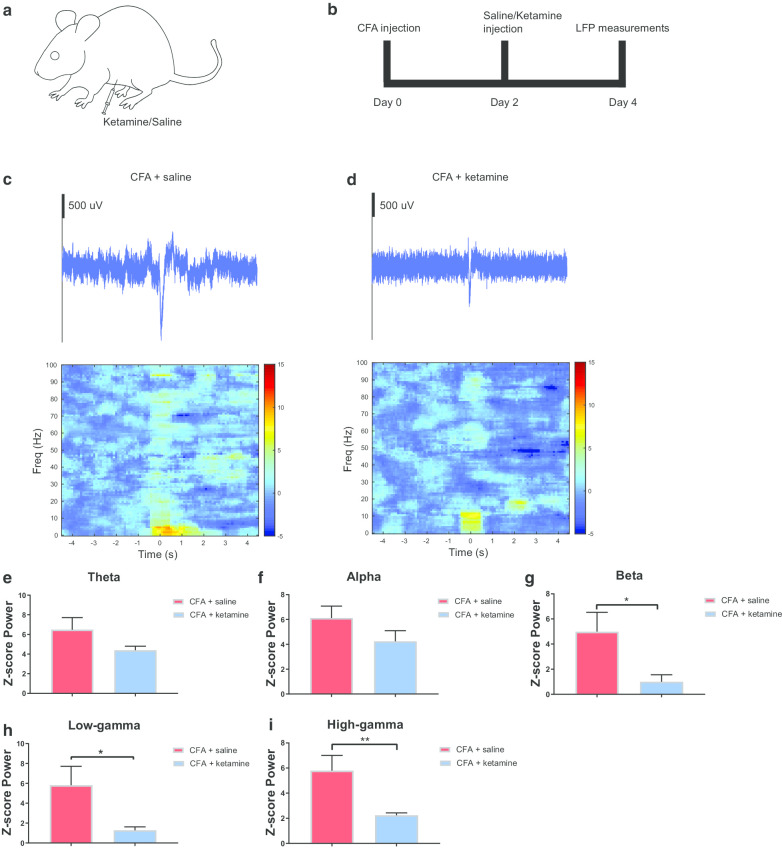
Ketamine inhibited the enhancement of high-gamma band power in CFA-treated rats. **a** Rats received either a ketamine or saline intraperitoneal injection. **b** Timeline for CFA, saline, and ketamine injections. **c** Example of trial-averaged LFP raw trace, in the chronic pain condition two days after saline injection. The panel directly below the raw trace shows trial-averaged time–frequency spectrum, with time 0 denoting the time of pinprick stimulation. **d** Example of trial-averaged LFP raw trace, in the chronic pain condition two days after ketamine injection. The panel directly below the raw trace shows trial-averaged time–frequency spectrum, with time 0 denoting the time of pinprick stimulation. **e** Z-score power in the theta frequency band (4–8 Hz) two days after saline or ketamine injection. **f** Z-score power in the alpha frequency band (8–15 Hz) two days after saline or ketamine injection. **g** Z-score power in the beta frequency band (15–30 Hz) two days after saline or ketamine injection (p = 0.0229, unpaired t-test). **h** Z-score power in the low-gamma frequency band (30–60 Hz) two days after saline or ketamine injection (p = 0.0267, unpaired t-test). **i** Z-score power in the high-gamma frequency band (60–100 Hz) two days after saline or ketamine injection (p = 0.0086, unpaired t-test). Error bars represent SEM. *p < 0.05; **p < 0.01

CFA-treated rats were administered two days later, intraperitoneally, with either saline (control) or ketamine (Fig. 3a, b). A sub-anesthetic dose of ketamine (10 mg kg^−1^) was chosen, based on previous studies [45–47]. The saline-treated rats demonstrated a sustained increase in power in the high-gamma region, as seen in the trial-averaged LFP spectrogram (Fig. 3c). In contrast, ketamine-treated rats showed a decrease in activity in the high-gamma band, as seen in the trial-averaged LFP spectrogram (Fig. 3d). Similar to rats in the pre-CFA state, the trial-averaged LFP raw trace for the control group, saline-treated rats, showed a larger peak compared with the trial-averaged LFP raw trace for ketamine-treated rats (Fig. 3c, d). We also examined the power in other frequency bands, and found no statistical significance in the theta or alpha frequency bands (Fig. 3e, f). We did observe, however, a statistically significant decrease in power in the beta and low-gamma bands (p = 0.0229 and p = 0.0267, respectively, unpaired t-test, Fig. 3g, h). The power reduction in the high-gamma band was substantial, as the power returned to the baseline value prior to CFA injection (p = 0.0086, unpaired t-test, Fig. 3i). These data indicate that ketamine has the potential to reduce the power in the beta and low-gamma bands in CFA-treated rats. Specifically in the high-gamma band, ketamine appears to inhibit the increase of power associated with chronic pain.

### Ketamine provides sustained inhibition of LFPs in the high-gamma band

**Fig. 4:**
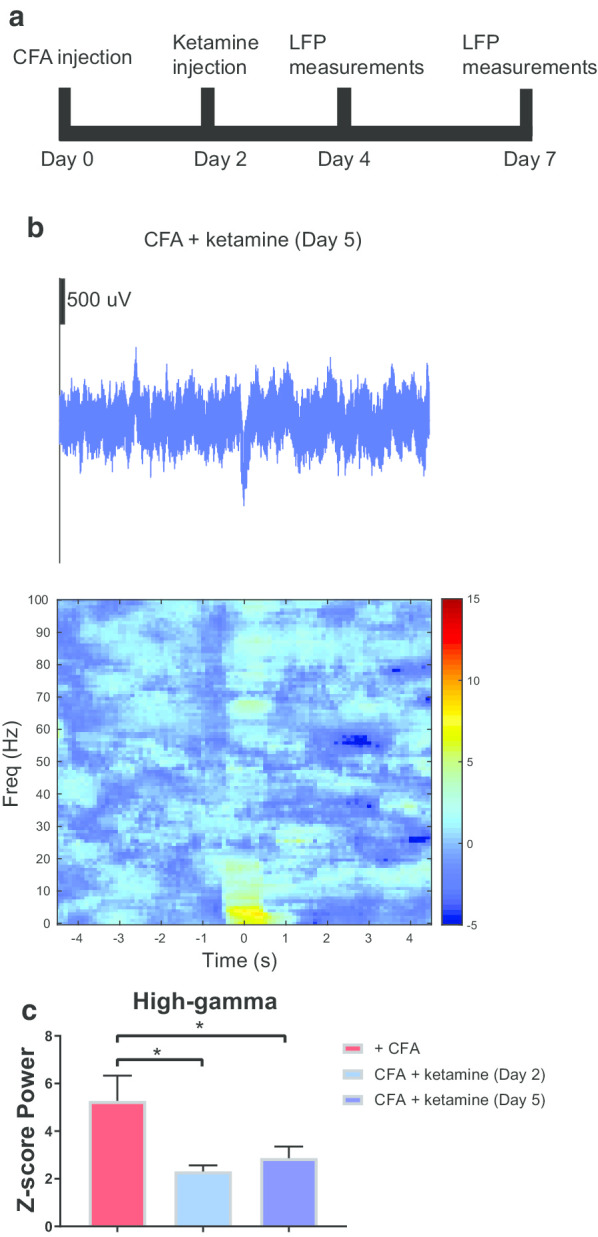
Ketamine had a long term effect of inhibition on the high-gamma band power in the ACC in chronic pain state. **a** Timeline for CFA and ketamine injections. **b** Example of trial-averaged LFP raw trace, in the chronic pain condition five days after ketamine injection. The panel directly below the raw trace shows trial-averaged time–frequency spectrum, with time 0 denoting the time of pinprick stimulation. **c** Z-score power in the high-gamma frequency band (60–100 Hz) post CFA injection, two days after ketamine injection, and five days after ketamine injection (p = 0.0140 and p = 0.0322, respectively, One-way ANOVA with Dunnett’s multiple comparisons test). Error bars represent SEM. *p < 0.05

Previous research suggests ketamine produces antidepressant effects that can last multiple days after a single administration [13, 15, 48–50]. To test whether a single injection of ketamine could reduce ACC activity over a sustained period of time, we measured LFPs 5 days after the ketamine injection (Fig. 4a). Five days after the ketamine injection, the trial-averaged spectrogram demonstrates a sustained power decrease in the high-gamma band (Fig. 4b). Furthermore, the peak of the trial-averaged LFP raw trace was consistent with the peaks of pre-CFA and day two ketamine-treated traces (Fig. 4b). Furthermore, we analyzed LFP power quantitatively and found a statistically significant difference comparing Day 2 and Day 5 after ketamine injection to the pre ketamine chronic pain state (p = 0.0140 and p = 0.0322, respectively, One-way ANOVA using Dunnett’s multiple comparisons, Fig. 4c). These data indicated that a single dose of ketamine is able to inhibit an increase in power in the high-gamma band and restore CFA-treated rats to a pre-CFA level of activity for up to five days. Thus, for rats in the chronic pain state, LFPs in the ACC demonstrate a sustained reduction in population activity due to a single ketamine injection.

## Discussion

This study is aimed at investigating LFP responses in the ACC to noxious stimulations in the chronic pain state and the effect of ketamine on these responses. We have demonstrated that chronic inflammatory pain causes an increase in spectral power in the high-gamma band in the ACC, and that ketamine reduces this abnormal increase in high-gamma power for at least 5 days.

Changes in gamma oscillations in acute and chronic pain states have been studied in both animal models and human subjects, and our observed increase in high-gamma band power is consistent with previous results [51–56]. Our finding that chronic pain caused increased power in the high-gamma band specifically in the ACC is consistent with another previous thermal pain study [57]. Interestingly, however, in the current study, we did not observe power enhancement in the theta band, in contrast to what was found with earlier studies in thermal stimulation [57, 58]. One reason for this discrepancy may be that mechanical pinprick stimulations, as opposed to thermal stimulations, elicit a greater theta band power response, thereby occluding further changes in the chronic pain conditions. This hypothesis is indirectly supported by a previous thermal pain study, which demonstrated a smaller change in CFA-induced theta band power elicited by high-intensity noxious stimuli compared with low-intensity noxious stimuli [57]. Importantly, different peripheral sensory neurons may respond to different intensities of mechanical and thermal stimulations [59]. A-delta fibers conduct quickly in response to highly noxious stimuli, particularly highly noxious mechanical stimuli [60]. In contrast, while C-fibers respond to both mechanical and thermal stimuli, they conduct slowly and play a greater role in distinguishing slower changes in temperature and resulting thermal pain intensity [61, 62]. At the molecular level, different mechanosensory or thermal receptors on these pain fibers also respond with different kinetic and thermodynamic profiles [63, 64]. Thus, changes in cortical oscillations may also reflect changes in peripheral, spinal and thalamic nociceptive pathways, which are complex and diverse in nature.

The role of ACC in pain processing, particularly the processing of affective component of pain, has been well documented [25, 31, 65–71]. Our results here further support these roles. Meanwhile, in a previous study, rats in the chronic pain state that received a ketamine injection demonstrated decreased pain aversion compared to control rats [36]. This study further demonstrated that ketamine likely reduced the affective component of pain by suppressing hyperactivity of ACC neurons in the chronic pain state [36]. In another study, ketamine has been shown to reduce depression-like behaviors in the chronic neuropathic pain state as well [46]. While our current results do not directly link high-gamma changes in the ACC with the analgesic properties of ketamine, in the context of these previous results they lend additional support to the role of ketamine in shaping functional connectivity involving ACC neurons. Future experiments that directly link LFP changes with behavioral observations would further our understanding of the impact of ketamine on the ACC in chronic pain states.

An important pathological feature of chronic pain is an amplified aversive response to noxious stimuli in an anatomically nonspecific manner, as found in conditions of fibromyalgia and persistent postoperative pain [19, 21, 72, 73]. Previous animal studies have shown chronic pain can alter synapses and circuits in the cerebral cortex, especially in the ACC [67, 70]. In a previous study analyzing the effect of ketamine on neuronal firing activity, ACC neuronal firing rates increased in the chronic pain state and returned to naïve levels after a single sub-anesthetic dose of ketamine [36]. Interestingly, in the present study we found that ketamine could reduce the ACC high-gamma band power enhancement induced by chronic pain. The time scale of gamma waves (10–20 ms) corresponds to the temporal window of spike timing-dependent plasticity involved in shaping synaptic connections [74, 75]. Thus, the high-gamma band power inhibition observed in the current study indicates that ketamine has the potential to reverse abnormal neuronal plasticity developed in the chronic pain state. Furthermore, the power reduction in the high-gamma band in the ACC lasted five days, which is consistent with the known time scale for the effect of ketamine on mood and pain [15–18, 46]. Previous studies have shown that inhibition of the *N*-methyl-d-aspartate receptors (NMDARs) by ketamine can increase brain derived neurotrophic factor (BDNF) expression in the hippocampus and prefrontal cortex, important regions for pain and mood regulation [18, 76]. In addition, ketamine can also upregulate mTORC1, a translational regulator, to promote the expression of specific synaptic proteins in the cortex [45, 77, 78]. Through this mechanism, ketamine can cause persistent increase in α-amino-3-hydroxy-5-methyl-4-isoxazolepropionic acid (AMPA) receptor-mediated neurotransmission to produce sustained antidepressant effects [79–84]. Thus, at the molecular level, its impact on central glutamate signaling underlies the long-lasting effects of ketamine on pain behaviors and may also mediate its effect on high-gamma oscillations in the ACC. Previous studies have also found that ketamine can decrease the power in low-frequency bands [85–88], and changes in the low-gamma and beta region have in fact been associated with altered glutamatergic levels in the ACC [89]. Interestingly, we also observed decreases in the power of low-gamma and beta bands after ketamine administration in the current study. Thus, ketamine has the potential to alter functional connectivity in the brain through widespread changes in the power spectra.

In our study, ketamine produced anti-aversive effects through actions on the ACC. However, as an antagonist of NMDARs, ketamine is likely to modulate brain state and change neuronal plasticity in many areas, since NMDARs play a critical role in synaptic plasticity throughout cortical and subcortical areas [90–95]. For example, a recent animal study has shown that ketamine can relieve symptoms of depression by blocking bursting in the lateral habenula [49]. In addition, ketamine is able to produce rapid antidepressant responses by inducing prefrontal cortex synaptogenesis and reversing the synaptic deficits caused by chronic stress [96]. A recent study has shown that ketamine is capable of activating GABAergic neurons in the central amygdala in both acute and chronic pain states [97]. Other regions such as the insular cortex, periaqueductal gray, and nucleus accumbens may also be involved in the anti-aversive effects of ketamine. Thus, future studies should focus on examining LFPs from multiple regions to obtain a comprehensive overview of the anti-aversive effects of ketamine at a broader pain network level.

Movement artifacts constitute a technical challenge for LFP recordings in freely moving animals. Multiple steps were taken in the present study to minimize movement artifacts. First, we removed all trials with abnormally high amplitudes, most likely resulting from unrelated or sudden reflexive motion. Second, we verified each trial with a video recording and removed the trial if unrelated movement was observed. Lastly, if a majority of channels contained noisy signal the trial was excluded, and if a majority of all trials in a given session were noisy, the entire session was excluded. In terms of final data analysis, noisy signals as the result of movement are expected to demonstrate an increase in power. Since our data showed a decrease in high gamma band power in the chronic pain state, noisy trials as the result of movement artifact likely did not significantly impact our findings. Nevertheless, to further minimize movement artifacts, future LFP recordings can be done with simultaneous neck electromyography recordings and motion monitoring with an ultra high-speed camera.

Other areas of limitation of this study include the use of a single dose of ketamine and a single chronic pain model. In addition, this study primarily addresses neural changes in response to evoked pain. Chronic pain, however, also includes spontaneously occurring pain episodes. Thus, future studies examining different dosages of ketamine, in the context of multiple pain behaviors, including spontaneous pain behaviors, in additional pain models are needed to further elucidate the cortical mechanisms of ketamine in treating chronic pain.

LFPs bear resemblance to electroencephalogram (EEG) signals, allowing studies of LFPs in animals to be translated to studies of EEG signals in humans [98]. This is supported by findings of increased gamma oscillations and quantification of pain perception in the somatosensory cortex in response to noxious stimuli [99, 100]. Recent technical development allows a single EEG electrode to record temporal–spectral neural patterns over single-trial stimulations and provide information about neuronal responses and sensitivity to pain [101]. Thus, if high frequencies of LFPs can be used as proxies for assessing the neuronal output, our system of inquiry can be potentially translated to EEG signals to introduce a noninvasive method to measure extracellular activity and objectively record and analyze human response to chronic pain.

In conclusion, we found that chronic pain increased power in the high-gamma band in the ACC of rats. A single sub-anesthetic dose of ketamine was able to rescue activity in the high-gamma band to reverse the changes induced by CFA. Furthermore, these effect on high-gamma band power lasted up to 5 days after ketamine injection. These findings suggest ketamine can impact network neuronal activity and cortical plasticity in the chronic pain state.

## Methods and materials

### Experimental animals

All procedures in this study were approved by the New York University School of Medicine (NYUSOM) Institutional Animal Care and Use Committee (IACUC) as consistent with the National Institute of Health (NIH) Guide for the Care and Use of Laboratory Animals to ensure minimal animal use and discomfort. Animals consisted of male Sprague–Dawley rats, 250 to 300 g each upon arrival, purchased from Taconic Farms (Albany, NY). Animals were housed at the Mispro Biotech Services Facility in the Alexandria Center for Life Science in a controlled environment, monitoring temperature, humidity, and 12 h (6:30 A.M. to 6:30 P.M.) light–dark cycle. Food and water were available ad libitum. Animals were given on average 14 days to adjust to the new environment before beginning experiments.

### Drugs

Rats were injected with 0.06 mL of CFA (*Mycobacterium tuberculosis*, Sigma-Aldrich) to induce inflammatory pain in the injected paw and initiate a chronic pain model. CFA was initially suspended in an oil-saline (1:1) emulsion and subsequently injected subcutaneously into the plantar aspect of the hind paw, ipsilateral to location of recording tetrodes and opposite the paw receiving pinprick stimulations. Ketamine-treated rats received one 0.5 mL 10 mg kg^−1^ injection of ketamine hydrochloride (Ketaset), purchased from Zoetis, intraperitoneally. The control group received an equal volume of saline injected intraperitoneally.

### Electrode implant and surgery

Two twisted 12.7 µm polyimide-coated microwires (Sandvik) were used to construct the stereotrodes. The stereotrodes were then mounted in a VersaDrive8 (Neuralynx), similar to other experiments, and dental cement was used to secure the drive to the skull screws [70, 102]. To reduce electrode impedances to 100–500 kΩ, electrode tips were plated with gold. In order to implant stereotrodes, the skull was exposed allowing a craniotomy to be performed over unilateral anterior cingulate cortex (AP + 2.5–3.5 mm, ML 0.8–1.8 mm). Isoflurane (1.5–2%) was used to anesthetize rats during implantation. With the tip angled 10° toward the midline, the electrode bundle was lowered at DV 1.6 mm. The average recovery time post-surgery, before neural recordings, was 1 week.

### In vivo electrophysiological recordings

Rats with electrode implants were place in a recording chamber over a mesh table and given 30 min prior to the onset of stimulations to adjust to the environment [70]. After the initial adjustment period, approximately 30 trials with variable inter-trial intervals (approximately 1 min) were conducted per session in free-moving rats. Inter-trial intervals were set between trials to avoid sensitization. Each trial consisted of a noxious simulation by pricking using a 27-gauge needle administered to the plantar surface of the hind paw contralateral to location of electrode implant and concluded by paw withdrawals. Throughout all sessions, no physical damage or behavioral sensitization to the paw was discerned. All sessions were recorded with a 120 fps video camera (DMK23U, image source).

### Neural data collection and preprocessing

Stereotrodes were lowered 60 µm interval each day before recording. During sessions, neural activity and signals before, during, and after, pinprick stimulations were recorded at a sample rate of 40 kHz using acquisition equipment (OmniPlex D with Digital Headstage Processor, Plexon). Raw data of LFPs was digitally filtered with a bandpass filter between 0.3 and 300 Hz and then down-sampled to 1 kHz.

### Histocytochemistry

Isoflurane was used to anesthetize rats whom were then transcardially perfused with ice-cold phosphate-buffered saline (PBS) and paraformaldehyde (PFA). Brains were fixed in PFA overnight and subsequently transferred for 3 days to 30% sucrose in PBS to equilibrate [103]. Microm HM252 Cryostat (Thermo Fisher Scientific) was used to collect 20 µm coronal sections. These sections were then washed in PBS and covered with a Vectashield mounting medium. If the section contained an electrode it was stained with cresyl violet or hematoxylin and eosin stain and analyzed with a Nikon eclipse 80i microscope with a DS-U2 camera head. Animals with incorrect electrode placement were excluded.

### Data preprocessing

Multi-channel LFP signals were saved from Plexon system, and we preprocessed the raw data to remove noisy trials. If sessions had been spike sorted, three channels were chosen based on those that had the greatest signal to noise ratio. If sessions had not been sorted, all channels with available LFP data were used. These denoised single-channel LFP signals were then utilized for subsequent spectrum analyses.

Multiple steps were taken in the present study to minimize movement artifacts. First, we removed all trials with abnormally high amplitudes, most likely resulting from unrelated or sudden reflexive motion. Second, we verified each trial with a video recording and removed the trial if unrelated movement was observed. Lastly, if a majority of channels contained noisy signal the trial was excluded, and if a majority of all trials in a given session were noisy, the entire session was excluded.

### Spectrum analysis

We opted for a multitaper method to compute spectrum analysis of LFP signals. The multitaper method is a spectral analysis technique to reduce bias/variance of spectral estimates [104]. It does this by pre-multiplying the data with orthogonal tapers, known as Slepian functions. We set a half-bandwidth parameter W, which defines the dimensions of the moving window to be [− W, W]. In order to ensure the Slepian taper functions were contained in frequency and have bias reducing characteristic, W was set to be greater than 1/T, where T indicates the temporal duration. The multitaper method and spectrum analysis was computationally generated with the Chronux toolbox [105]. The Chronux toolbox is an open source data analysis software (https://chronux.org). Spectrograms were generated using the function ‘mtspecgramc’. The parameters for mtspecgramc was [TW K], where TW = 3 and K = 2 × TW-1 = 5. TW is the time-bandwidth product and K is the number of tapers. A single vector was produced by summing all power values in each frequency band (theta 4–8 Hz, alpha 8–15 Hz, beta 15–30 Hz, low-gamma 30–60 Hz, high-gamma 60–100 Hz). Then for each frequency band we computed the Z-score of power related to the baseline period [− 4.5, − 0.5] s before pinprick simulation, and computed the average over the stimulus range [− 0.5 1.0], where 0 denotes the time of pinprick stimulation, to produce a single power value. Estimated power values were then averaged over all selected channels to produce one estimated power value for the given session for a single rat. If the Z-score was positive, it demonstrated an increase in power at the specific frequency band(s).

### Statistical analysis

To compare Z-score power values of rats before CFA injection with rats after CFA injection, we used a Paired t-test. To compare saline injections with ketamine administrations in CFA-treated rats, we used an unpaired t-test. To compare ketamine administration over multiple days in CFA-treated rats, we used a One-way ANOVA test with post-hoc Dunnett’s multiple comparisons. We reported the mean ± SEM statistics in LFP power**.** For all tests, a *p* value < 0.05 was considered statistically significant. All data was analyzed using the GraphPad Prism Version 7 software (GraphPad) and MATLAB (MathWorks).

## Data Availability

All the data and code are available from the corresponding author on reasonable request.

## Abbreviations

CFA: Complete Freund’s adjuvant
LFPs: local field potentials
ACC: anterior cingulate cortex
NMDARs: *N*-methyl-d-aspartate receptors
EEG: electroencephalogram
AMPA: α-amino-3-hydroxy-5-methyl-4-isoxazolepropionic acid
